# Measuring students’ approaches to learning in different clinical rotations

**DOI:** 10.1186/1472-6920-12-114

**Published:** 2012-11-15

**Authors:** Ova Emilia, Leah Bloomfield, Arie Rotem

**Affiliations:** 1 Faculty of Medicine, Universitas Gadjah Mada, Jl. Farmako, Sekip Utara, Yogyakarta, Indonesia; 2 School of Public Health and Community Medicine, UNSW, Sydney, Australia

**Keywords:** Approaches to learning, Study Process Questionnaire (SPQ), Learning process, Clinical teaching

## Abstract

**Background:**

Many studies have explored approaches to learning in medical school, mostly in the classroom setting. In the clinical setting, students face different conditions that may affect their learning. Understanding students’ approaches to learning is important to improve learning in the clinical setting. The aim of this study was to evaluate the Study Process Questionnaire (SPQ) as an instrument for measuring clinical learning in medical education and also to show whether learning approaches vary between rotations.

**Methods:**

All students involved in this survey were undergraduates in their clinical phase. The SPQ was adapted to the clinical setting and was distributed in the last week of the clerkship rotation. A longitudinal study was also conducted to explore changes in learning approaches.

**Results:**

Two hundred and nine students participated in this study (response rate 82.0%). The SPQ findings supported a two-factor solution involving deep and surface approaches. These two factors accounted for 45.1% and 22.5%, respectively, of the variance. The relationships between the two scales and their subscales showed the internal consistency and factorial validity of the SPQ to be comparable with previous studies. The clinical students in this study had higher scores for deep learning. The small longitudinal study showed small changes of approaches to learning with different rotation placement but not statistically significant.

**Conclusions:**

The SPQ was found to be a valid instrument for measuring approaches to learning among clinical students. More students used a deep approach than a surface approach. Changes of approach not clearly occurred with different clinical rotations.

## Background

The concept of approach to learning has been studied extensively because it is strongly related to students’ level of understanding and learning outcomes. In general, three different approaches have been described: deep, surface and strategic [1-3]. Students who adopt a deep approach are predominantly motivated by an interest in learning for its own sake and an interest in the subject material. They attempt to understand the underlying structure and meaning, examine evidence critically, use it cautiously and actively relate new information to previous knowledge. Students who adopt a surface approach are predominantly motivated either by a desire simply to complete the course or by a fear of failure. Their intention is to fulfill the course requirements by memorizing and reproducing specific facts or pieces of disconnected information for examinations. They tend to be anxiously aware of assessment requirements and prefer to restrict learning to a defined syllabus and specified tasks. Students who adopt a strategic/achieving approach are predominantly motivated by the achievement of high grades and a sense of competition. Their main intention is to be successful, and they are prepared to use any means necessary, depending on what they feel would produce the most successful results.

Several instruments have been developed to measure the behavioral and conceptual processes in which students engage while learning. These include the Inventory of Learning Processes, the Approaches to Studying Inventory and the Study Process Questionnaire (SPQ) [4]. This study focused on the SPQ.

The SPQ was developed from the earlier 10-scale Study Behaviour Questionnaire (SBQ), which was conceived within an information-processing framework. The SBQ initially contained 72 items, but was later extended to 80 items covering 10 subscales. In the subsequent SPQ, some of the items in the SBQ were reclassified and the 10 subscales were relabeled [1]. It was demonstrated that these relabeled subscales could be subsumed under three second-order factors interpreted as the reproducing, internalizing and organizing dimensions of students’ learning. Although these were intended to encompass the values, motives and cognitive strategies associated with particular aspects of learning, they are broadly comparable with the surface, deep and strategic approaches that had been defined earlier. Ultimately, Biggs [5] reduced the SPQ to seven items in each of six subscales reflecting the respondents’ motives and strategies within each of the three major dimensions, and renamed them as surface, deep and achieving approaches to learning to bring the terminology into line with that of other researchers (Table 1).

**Table 1:** Three prototypical approaches to learning

**Approach**	**Motive**	**Strategy**
Surface	Extrinsic: avoid failure but don’t work too hard	Focus on selected details and reproduce accurately
Deep	Intrinsic: satisfy curiosity about topic	Maximize understanding: read widely, discuss, reflect
Achieving	Achievement: compete for highest grade	Optimize organization of time and effort (study skills)

In 1992, Biggs [5] summarized work with the 3P (presage, process, product) model using the SPQ, focusing on students’ motives and strategies for learning. These motives and strategies have been examined in various contexts, including cross-cultural comparisons, the language medium of instruction, teaching and learning environments, professional and staff development, and factor structure and the dimensionality of subscales [6-8].

When using the SPQ to monitor teaching/learning environments, the role of the scales related to achieving is not as evident as that of the deep and surface scales. Indeed, the achieving motive and strategy differ from the deep and surface motives and strategies from the outset [5]. Whereas deep and surface strategies describe the way students engage in a task, the achieving strategy refers to how the student organizes when and where the task will be engaged, and for how long. Factor analyses usually associate the achieving motive and strategy with the deep approach, but depending on the subject and teaching conditions, achieving-related scores sometimes have a bearing on the surface approach. Using confirmatory factor analysis, the SPQ is most conveniently described in terms of two factors – deep and surface – with achieving motive and strategy subscales aligned with both of these factors [7,9].

Responding to the need for a shorter, two-factor version of the SPQ that addresses deep and surface approaches only and can be administered quickly and easily by a regular teacher, Biggs and colleagues [10] developed a simple version comprising two factors (deep and surface) with 20 items. This version of the questionnaire has acceptable Cronbach’s alpha reliability values (0.64 for surface approach and 0.73 for deep approach).

Concerning medical students’ approaches to learning, studies have used several different instruments: Biggs’s SPQ, obtaining scores for surface, deep and achieving learning approaches; the Entwistle–Ramsden Lancaster Approaches to Studying Inventory, obtaining scores for reproducing, meaning and achieving orientations to learning; the Schmeck Inventory of Learning Process; and the Adelaide Diagnostic Learning Inventory for Medical Students [1-3,11-14].

These earlier studies investigated approaches to learning in the classroom setting. However, learning in the clinical setting during medical education is a different environment, and differences in learning are associated with department-related and student-related factors (one being the approach to learning) and interactions between them. Understanding the student factor is important in improving learning in the clinical setting [15-17]. In a recent qualitative study in a clinical learning context, three factors affecting students’ learning approaches were identified: clinical supervisors and supervision, stress and anxiety, and assessment [18]. The purpose of the present study was to assess the reliability and validity of the SPQ for use in clinical learning in medical education and also to determine whether learning approaches vary between rotations.

## Methods

### Participants

A cross-sectional quantitative study was conducted among students in years 5 and 6 who were undertaking clerkships at the Faculty of Medicine, Universitas Gadjah Mada, Indonesia. The clerkships comprise several rotations of varying length that all students should follow completely. For the study, the inclusion criterion was undergraduate medical students who were undertaking a clinical clerkship during the data collection period. Students in community medicine or public health rotations who were working outside the hospital setting were excluded.

The SPQ questionnaire had already been modified for the clinical context and was distributed to 255 students in the last week of their clerkship rotation. The students were asked to complete the questionnaire related to their current rotation and return it at the end of the week to their student coordinator, who then sent the questionnaires to the researcher. Only students who returned a completed questionnaire were included in the analysis.

A sample of students was selected to participate in a more extensive longitudinal study to explore changes in approaches to learning in three different rotations over a period of 30 weeks. Random sampling was not feasible. Thus, group randomization representing different rotations was used. This resulted in three groups from internal medicine, surgery and neurology that comprised 39 students. For this longitudinal study, a questionnaire survey after each rotation was conducted, and focus group discussions were held at the end of the three rotations.

### The instrument

The SPQ was selected from various instruments that measure students’ learning approaches. According to Biggs, a student’s learning approach is a function of both a motive and a strategy [1], with the motive influencing the learning and studying strategies that the student adopts. Although Biggs claimed that students’ approaches are relatively stable over time, several studies have demonstrated the contextual dependence of learning approaches [19,20]. In the present study, approaches to learning were determined in the context of studying in a clinical department. The original instrument comprised 42 items (six subscales) rated on a five-point Likert scale, with a value of five indicating that the statement was “always or almost always true of me” and one indicating that the statement was “never or only rarely true of me”. To apply the questionnaire to medical education, Hilliard [12] removed 18 items containing general or abstract statements that might interfere with the “specific” content sought in the medical education setting. The present study used items similar to those in Hilliard’s study. However, because of the clinical setting, 24 items in the Hilliard questionnaire were reworded to reflect the clinical teaching context. For example, item 4 in the original version (“I think browsing around is a waste of time, so I only study seriously what’s given out in class or in the course outlines”) was reworded to “I think that trying to be involved in all clinical activities is a waste of time, so I devote my effort to areas clearly identified in the objectives of this clerkship”. In some cases the adjustment was slight, with only the context being changed. For example, item 20 (“I usually become increasingly absorbed in my work the more I do”) was changed to “The more I am involved in clinical activities, the more absorbed I become in my work” [see Additional file 1.

### Statistical analysis

Descriptive statistics were used for the participants’ characteristics. Factor analysis using principal component analysis with oblimin rotation and internal consistency methods was conducted to examine the construct validity and the reliability of the scales. Correlation analysis among subscales and repeated measures analysis of variance were used for statistical comparisons. SPSS (17.0) software [21] was used for data analysis, and a statistically significant difference between means was set at the 0.05 level.

### Ethics

Ethical clearance was received from the Ethics Committee for Biomedical Research Involving Human Subjects, the Universitas Gadjah Mada, Yogyakarta. No funding was sought for the study.

## Results and discussion

Of 255 students, 209 returned the questionnaire (response rate 82.0%). Of these, 95 students were in their fifth year, and 114 were in their sixth year. Almost twice as many were female (134) as male (75). Most were not married (183). One-half (107) were housed in dormitories or home stays, about one-third (76) lived with parents and a small proportion lived with other relatives (17) or alone (9). It is typical for out-of-town students to live with relatives, in dormitories or in home stays.

The means and standard deviations for the subscales of students’ approaches to learning and estimates of internal consistency are presented in Table 2. The scores for the deep learning approach were higher than those for the surface or achieving approach. Preclinical students in medical school are considered to have to be very competitive, high achievers to obtain high marks in examinations that test a wide range of factual knowledge. However, once a student has passed the early part of the course, success in the clinical part is assumed. Student comments during focus group discussions suggested that they are less competitive in their clinical years than during the earlier, more theoretical part of the course. Hilliard found that senior students were more interested in learning for the sake of learning and in acquiring the skills of a competent physician [12]. Furthermore, in the departments involved in the present study, assessments tend to require explanation and comprehension (oral examinations, case presentations and written assignments) and not merely memory recall. This practice may require students to use more deep learning.

**Table 2:** Mean scores on the subscales of approaches to learning

**Approach**/**scale/item**	**Mean**	**SD**	**Cronbach**’**s alpha**
Surface approach	**21**.**61**	**5**.**23**	0.74
Surface motive	11.46	2.19	0.65
Surface strategy	10.73	2.23	0.65
Deep approach	**27**.**49**	**5**.**98**	0.83
Deep motive	14.13	2.18	0.78
Deep strategy	13.94	2.12	0.79
Achieving approach	**26**.**30**	**6**.**02**	0.84
Achieving motive	12.88	2.57	0.73
Achieving strategy	14.01	2.36	0.81

Several studies of medical students in their non-clinical years have used either the Biggs SPQ instrument to measure surface, deep and achieving learning approaches, or the Entwistle–Ramsden Lancaster Approaches to Studying Inventory to measure reproducing, meaning and achieving orientations to learning [4,22,23]. The scoring methods used, numbers of items and medical school classes differed between these studies. All studies except Martenson’s found deep or meaning scores to be the highest [23]. The Martenson study, conducted in a traditional medical school, reported surface scores as the highest.

The overall alpha value and the alpha value for each scale indicated a high level of internal consistency (0.91) comparable with that in other studies [7,10,12,24,25]. In the present study, there were only four items per subscale. This could have been the reason for the higher alpha reliability (range 0.65–0.84) compared with the range (0.60–0.75) reported in studies with seven items per subscale [9]. Compared with a similar questionnaire applied in medicine, the alpha reliability was higher (0.57–0.67 in Hilliard [12]). Factors that may have influenced the higher reliability in this study were number of items (this study used the shorter version) and homogeneity of the students (age, marital status, and living arrangement) who completed the questionnaire. These results validate use of the modified questionnaire for assessing approaches to learning in a clinical setting. Further studies with larger samples are recommended to investigate the applicability of the questionnaire in other clinical settings.

To evaluate the construct validity of the questionnaire, the six subscale scores were subjected to principal component factor analysis followed by an oblimin rotation to a simple structure. Eigenvalues greater than 1.0 were adopted as a criterion for determining the number of factors to be extracted. Two factors with eigenvalues above 1.0, which accounted for 67.7% of the variance, were obtained and are shown in Table 3. Examination of the screen test supported the two-factor solution.

**Table 3:** Factors resulting from the factor analysis of approaches to learning subscales

**Subscale**	**Factor 1**	**Factor 2**
Surface Motive	0.11	**0**.**84**
Surface Strategy	0.17	**0**.**76**
Deep Motive	**0**.**85**	0.16
Deep Strategy	**0**.**88**	0.12
Achieving Motive	0.53	**0**.**61**
Achieving Strategy	**0**.**85**	0.30

On the basis of Biggs’s original theory, there should be a three-factor solution identifying three learning approaches: deep, surface and achieving. However, subsequent research [9] suggested that there are only two solutions, deep and surface. The achieving subscales were usually found to either load on one of the other two factors or be divided between them. In 2001, Biggs and colleagues verified the revision of the SPQ with a two-factor structure [10]. The revised instrument assesses deep and surface approaches only and uses fewer (20) items. The present study also supports a two-factor solution, with the “achieving strategy” loaded on factor 1 (deep approach) and the “achieving motive” loaded on factor 2 (surface approach). These two factors explain 45.1% and 22.5%, respectively, of the variance. Further discussion therefore considers only the deep and surface approaches to learning.

The relationships between the two scales and their subscales are shown in Table 4. There was a positive correlation (.845; .887; and .872) between the deep approach and its three subscales and also between the surface approach and its subscales (.765; .719; and .756). The correlations were significant at the 0.01 or 0.05 level. Weak correlations between approaches and subscales show that the two approaches were dissimilar.

**Table 4:** **Correlation between approaches to learning scales** (**coefficient correlations**)

**Scales**	**SA**	**SM**	**SS**	**AM**	**DA**	**DM**	**DS**	**AS**
Surface approach/SA	1.00							
Surface motive/SM	0.765**	1.00						
Surface strategy/SS	0.719**	0.390**	1.00					
Achieving motive/AM	0.756**	0.363**	0.261**	1.00				
Deep approach/DA	0.343**	0.129	0.181**	0.430**	1.00			
Deep motive/DM	0.279**	0.069	0.136*	0.390**	0.845**	1.00		
Deep strategy/DS	0.243**	0.056	0.134	0.330**	0.887**	0.644**	1.00	
Achiev. strategy/AS	0.363**	0.203**	0.196**	0.396*	0.872**	0.567**	0.680**	1.00

**Correlation is significant at the 0.01 level (two-tailed).

*Correlation is significant at the 0.05 level (two-tailed).

A surface approach will work if a student is trying to memorize information for reproduction [1]. However, excessive use of the surface approach can lead to missing interconnections between elements, or the meanings and implications of what is learned. Students who use a surface approach tend to have an external locus of control (e.g. are highly oriented toward grades), study less and target their study toward material they believe will be included in the examination. Students who are less oriented toward grades have a deep approach to learning and study material in a broader sense.

The students’ approaches to learning did not change significantly as they rotated through the three departments involved in this study. Table 5 presents the repeated measures analysis of variance results.

**Table 5:** Comparison between approaches to learning scores in three medical departments

**Scales**	**Internal Med Mean (SD)**	**Surgery Mean (SD)**	**Neurology Mean (SD)**	**F, (df), p, eta**^**2**^
Surface Approach	36.14(5.35)	34.81(6.08)	35.05(4.14)	.39 (2,39) .55 .02
Surface Motive	11.90(2.19)	11.29(2.61)	11.67(1.76)	.42 (2,39) .86 .02
Surface Strategy	11.28(2.07)	10.62(2.50)	11.09(1.64)	.50 (2,39) .61 .03
Achieving Motive	12.95(2.56)	12.90(3.10)	12.28(2.10)	.45 (2,39) .64 .02
Deep Approach	42.71(4.83)	42.92(5.59)	40.81(4.91)	1.18 (2,39) .32 .06
Deep Motive	14.33(1.96)	14.19(2.27)	14.00(1.76)	.15 (2,39) .86 .01
Deep Strategy	14.48(1.78)	14.43(1.94)	13.48(1.86)	1.90 (2,39) .16 .09
Achiev. Strategy	13.90(2.07)	14.33(2.67)	13.33(2.48)	.94 (2,39) .40 .05

The small differences in students’ approaches in this study suggest that contextual or environmental variables as well as individual perceptions of these variables influence students’ use of deep or surface approaches to learning tasks. The lack of significant results in the longitudinal study is possibly attributable to the small sample used (n=39) or the short period of time between measurements (six weeks for neurology and 12 weeks for internal medicine and surgery).

Despite there being no significant differences, the pattern of fluctuating surface and deep approach scores across different departments supports the idea that students have individual combinations of approaches when facing different tasks in the departments. These have been called study “orchestrations” by Meyer [26] and imply that students use strategies flexibly, according to which is most appropriate to the learning environment. In the present study, students had higher deep approach scores in all three departments, which may be explained by their being senior, clinical students. Lindblom-Ylanne and Lonka [27] suggested that an increasingly high degree of deep-level learning is needed toward the end of medical studies, and indeed found that this occurred in their study.

## Conclusion

The findings of this study indicate that the internal consistency and factorial validity of the SPQ are comparable with those reported previously. Therefore, the questionnaire can be used with confidence to assess learning and study processes in the clinical context as well as across different learning cultures and educational systems. Further psychometric and validation studies are needed to enhance the usefulness of this instrument in educational and psychological research. The clinical students in this study had higher scores for deep learning. Being relatively close to graduation may have made them serious about learning and keen to acquire the skills of a competent physician. The longitudinal study fails to provide significant evidence that approaches to learning change according to rotation placement. Factors that might influence these results could be of interest in further studies.

## Competing interests

The author declares that they have no competing interests.

## Authors’ contributions

All authors participated in designing the study as initiated by OE, who managed all practical parts of the study and drafted the manuscript. LB and AR participated in finalizing the manuscript. All authors read and approved the final manuscript.

## Pre-publication history

The pre-publication history for this paper can be accessed here:

http://www.biomedcentral.com/1472-6920/12/114/prepub

## Supplementary Material

Additional file 1 **Approaches to Learning Questionnaire.** The questionnaire used in this study, which was based on previous work by Biggs et al. [10] and Hillard [12]. Click here for file
