# Supplementary material for: Measuring students’ approaches to learning in different clinical rotations
Source: BMC Med Educ. 2012 Nov 15;12:114. doi: 10.1186/1472-6920-12-114 (PMC3527326; doi:10.1186/1472-6920-12-114)
Supplement: Additional file 1 — Approaches to Learning Questionnaire. The questionnaire used in this study, which was based on previous work by Biggs et al. [10] and Hillard [12]. [file 1472-6920-12-114-S1.pdf]

## APPROACHES TO LEARNING QUESTIONNAIRE

It is recognized that students adopt different approaches to their study. This section is intended to explore your individual approaches. There are no right or wrong answers, you may adopted an approach which is more appropriate to you. Please indicate your agreement and disagreement with the following statements by circling the appropriate number.

**Key:** 1= this item is never or only rarely true of me
2= this item is sometimes true of me
3= this item is true of me about half the time
4= this item is frequently true of me
5= this item is always or almost always true of me

---

| | | | | | |
|---|---|---|---|---|---|
| 1. In choosing studying medicine, I was more concern about my chance of getting good job than my interest in the study of medicine. | 1 | 2 | 3 | 4 | 5 |
| 2. I feel compelled to seek evidence from the latest scientific literature rather than depend completely on my clinical supervisor knowledge. | 1 | 2 | 3 | 4 | 5 |
| 3. I want top grades in this attachment to enable me to select my preferred residency. | 1 | 2 | 3 | 4 | 5 |
| 4. I think that trying to be involved in all clinical activities is a waste of time, so I devote my effort to areas clearly identified in the objectives of this clerkship. | 1 | 2 | 3 | 4 | 5 |
| 5. While I am studying a new skill, I often think of real life situations in which I could apply what I have learned. | 1 | 2 | 3 | 4 | 5 |
| 6. I tend to summarize all clinical discussions with supervisors and include these as part of my notes on a topic. | 1 | 2 | 3 | 4 | 5 |
| 7. I tend to be discouraged by a poor mark on an exam and too worry about my ability to succeed in the next exam. | 1 | 2 | 3 | 4 | 5 |
| 8. Experiencing clinical activities in this department influence my values and views about society in which I live and my own philosophy of life. | 1 | 2 | 3 | 4 | 5 |
| 9. I would do any clinical or non-clinical tasks expected of me in this department as long as it would lead me to high grade. | 1 | 2 | 3 | 4 | 5 |
| 10. I learn the protocol of procedures by going over them many times until I can remember them by heart. | 1 | 2 | 3 | 4 | 5 |
| 11. I try to relate what I have learned in this department to my study in other departments. | 1 | 2 | 3 | 4 | 5 |

**Key:** 1= this item is never or only rarely true of me
2= this item is sometimes true of me
3= this item is true of me about half the time
4= this item is frequently true of me

5= this item is always or almost always true of me

12. I tend to study and practice clinical skills and knowledge consistently throughout the term and review regularly closer to the exams. 1 2 3 4 5

13. Whether I like it or not, I realize that I must do the clinical clerkship in order to graduate and get a well paid job. 1 2 3 4 5

14. My involvement in clinical activities can be as exciting to me as the best social or recreational involvement. 1 2 3 4 5

15. I see getting high grades in this attachment is part of a competitive game, and I play to win. 1 2 3 4 5

16. I generally restrict my clinical involvement and study to what is specifically set in the objectives as I think it is unnecessary to do anything extra. 1 2 3 4 5

17. I spend a lot of my free time finding out more about interesting topics which have been discussed in different clinical sessions. 1 2 3 4 5

18. I tend to complete the assignments given by my supervisors as soon as they are given. 1 2 3 4 5

19. Supervisors shouldn't expect students to spend too much time on areas of knowledge and skills which they do not intend to examine. 1 2 3 4 5

20. The more I am involved in clinical activities, the more absorbed I become in my work. 1 2 3 4 5

21. One of the most important considerations in choosing to study medicine is my confidence that I will be successful in getting top marks. 1 2 3 4 5

22. I tend to accept the suggestions and ideas of my supervisors and tend not question them in front of other students. 1 2 3 4 5

23. I find learning clinical skills and procedures interesting and I don't mind staying longer in the hospital to achieve better understanding. 1 2 3 4 5

24. After a clinical session I tend to reread my notes to ensure that they are legible and I can understand them. 1 2 3 4 5
